# Clinicopathological Features, Tumor Localization and Treatment Outcomes in Paraganglioma: A Single-Center Medical Oncology Cohort

**DOI:** 10.3390/diagnostics16162516

**Published:** 2026-08-10

**Authors:** Hatice Asoglu, Esra Asarkaya, Abdurrahman Aykut, Gunes Dorukhan Cavusoglu, Yasemin Aydinalp Camadan, Irem Kolsuz Turker, Hacer Demirkose, Tolga Koseci, Ertugrul Bayram, Gamze Akkus, Ramazan Asoglu, Seyda Erdogan, Ismail Oguz Kara

**Affiliations:** 1 Department of Medical Oncology, Faculty of Medicine, Çukurova University, Adana 01330, Türkiye; 2 Department of Medical Oncology, Şanlıurfa Mehmet Akif İnan Education and Research Hospital, Şanlıurfa 63300, Türkiye; 3 Department of Endocrinology, Faculty of Medicine, Çukurova University, Adana 01330, Türkiye; 4 Department of Communicable Diseases and Early Warning, General Directorate of Public Health, Ministry of Health, Ankara 06430, Türkiye; 5 Department of Cardiology, Yalova State Hospital, Yalova Provincial Health Directorate, Yalova 77200, Türkiye; 6 Department of Pathology, Faculty of Medicine, Çukurova University, Adana 01330, Türkiye

**Keywords:** paraganglioma, recurrence-free survival, Ki-67, GAPP, tumor localization, medical oncology, prognosis, peptide receptor radionuclide therapy

## Abstract

**Background/Objectives:** Paragangliomas (PGLs) are rare neuroendocrine tumors, and data describing them from a medical oncology perspective are limited. We characterized clinicopathological features, tumor localization, and treatment outcomes. **Methods:** We retrospectively analyzed 43 patients with PGL at a single medical oncology department. The primary endpoint was recurrence-free survival (RFS), defined as time to first recurrence or death from any cause; overall survival (OS), objective response rate (ORR), disease control rate (DCR), and prognostic associations were secondary. **Results:** Median age was 44 years, 60.5% were female, and tumors were sympathetic (extra-adrenal) in 55.8% and parasympathetic (head and neck) in 39.5%. After a median follow-up of 116.8 months, 40 patients (93.0%) underwent resection, among whom 14 RFS events occurred (13 recurrences, 1 unrelated death). Median RFS and OS were not reached (60-month RFS, 67.1%; 120-month OS, 83.3%). Among nine patients receiving first-line systemic therapy (eight response-evaluable), ORR was 12.5% and DCR 37.5%. In exploratory univariable analysis, a Ki-67 index ≥ 3% correlated with recurrence (time-averaged hazard ratio, 4.53; 95% CI, 1.55–13.19; *p* = 0.006), alongside an R1 resection margin (not significant after Bonferroni correction). **Conclusions:** These findings may support further evaluation of Ki-67 within risk-adapted surveillance but do not replace germline testing.

## 1. Introduction

Paragangliomas (PGLs) are rare neuroendocrine tumors that arise from chromaffin cells of the extra-adrenal autonomic paraganglia, in contrast to pheochromocytomas (PCCs), their counterparts within the adrenal medulla [1]. PGLs are classified according to their autonomic origin and anatomical distribution: sympathetic PGLs develop along the paravertebral and para-aortic axes of the thorax, abdomen, and pelvis and commonly secrete catecholamines, whereas parasympathetic PGLs of the head and neck arise along the glossopharyngeal and vagal nerves and are usually nonsecreting [1,2]. Abdominal sympathetic PGLs represent a substantial proportion of extra-adrenal tumors and are frequently managed across surgical and oncological services [3]. Within the broader family of pheochromocytomas and paragangliomas (PPGLs)—which has an estimated annual incidence of 0.2–0.8 per 100,000 and an approximately equal sex distribution—PGLs account for roughly one-fifth of cases [2].

Catecholamine excess in secretory tumors accounts for the classic presentation of paroxysmal hypertension, headache, palpitations, and diaphoresis, whereas nonsecretory head and neck PGLs more often present as a painless mass, and an increasing number of tumors are detected incidentally or through genetic surveillance [2,4]. PPGLs exhibit one of the highest rates of hereditary predisposition among human neoplasms, with more than 20 susceptibility genes characterized to date and a causal germline variant identified in approximately 40% of patients [4,5]; genetic testing is therefore recommended in all patients [6]. On the basis of their transcriptional programs, these tumors are grouped into three molecular clusters: a pseudohypoxia-associated cluster 1 (encompassing *SDHx*, *VHL*, and *EPAS1*), a kinase-signaling cluster 2 (*RET*, *NF1*, *MAX*, *TMEM127*, and *HRAS*), and a Wnt-signaling cluster 3 (*MAML3* and *CSDE1*) [5,7,8]. Cluster assignment correlates with biochemical phenotype, anatomical distribution, and metastatic risk; *SDHx*-related disease in particular is enriched among extra-adrenal PGLs and is increasingly used to inform functional imaging, surveillance, and treatment selection [7,9].

All PPGLs are now regarded as neoplasms with metastatic potential; accordingly, the current World Health Organization classification no longer applies the historical benign–malignant dichotomy and defines metastatic disease by tumor dissemination to sites where chromaffin tissue is normally absent [10]. PGLs carry a substantially higher metastatic risk than PCCs and tend to metastasize earlier, with extra-adrenal location and germline *SDHB* variants conferring the greatest risk; the bones, regional lymph nodes, liver, and lungs are the most frequent sites [11,12]. Because no single histological feature reliably separates indolent from aggressive tumors, composite tools such as the Pheochromocytoma of the Adrenal gland Scaled Score (PASS) and the Grading of Adrenal Pheochromocytoma and Paraganglioma (GAPP)—the latter incorporating the Ki-67 proliferation index and catecholamine phenotype—have been proposed for risk stratification, although their reproducibility and prognostic value remain debated [13,14,15,16]. Larger tumor size, a dopaminergic phenotype, and incomplete resection have additionally been linked to a more aggressive clinical course [17]. The prognosis of metastatic disease is heterogeneous but frequently unfavorable, with a reported 5-year survival of approximately 60% [12,18].

Therapeutic options for advanced or metastatic disease are limited and supported largely by retrospective experience [18,19]. Systemic strategies include combination chemotherapy with cyclophosphamide, vincristine, and dacarbazine (CVD), temozolomide, tyrosine kinase inhibitors such as sunitinib, and radionuclide therapies with iodine-131-metaiodobenzylguanidine ([131I]-MIBG) or somatostatin-receptor-directed peptide receptor radionuclide therapy (PRRT) [19,20]; PRRT can achieve durable clinical and biochemical responses [21]. In therapy-naive patients, a substantial proportion achieve disease stabilization, supporting active surveillance in selected cases [22]. Objective response and disease-control rates differ considerably across these modalities and appear to be modulated by genotype, with *SDHB*- and *SDHD*-mutated tumors showing comparatively favorable responses to CVD in some series [19,23].

Despite this expanding therapeutic landscape, most published series originate from endocrinology and surgical settings and emphasize perioperative preparation and biochemical control, whereas data describing PGLs from a medical oncology standpoint—centered on advanced disease and systemic treatment outcomes—remain comparatively scarce. To help address this gap, we retrospectively examined a single-center medical oncology cohort of patients with paraganglioma, characterizing their clinicopathological features, tumor localization, and treatment outcomes.

## 2. Materials and Methods

### 2.1. Study Design and Setting

This single-center, retrospective cohort study was conducted at the Department of Medical Oncology, Çukurova University Faculty of Medicine, Adana, Türkiye. Consecutive patients with paraganglioma diagnosed between June 2007 and September 2025 and referred to and followed at the medical oncology department were identified from institutional records. The study was approved by the Çukurova University Faculty of Medicine Research Ethics Committee (meeting no. 165, decision no. 25; 3 April 2026) and performed in accordance with the Declaration of Helsinki; the requirement for written informed consent was waived because of the retrospective, anonymized design. The data cutoff date was 30 April 2026.

### 2.2. Patients

Eligible patients had a diagnosis of paraganglioma established by histopathology and/or characteristic cross-sectional and functional imaging. Both parasympathetic (head and neck) and sympathetic (extra-adrenal) paragangliomas were included, irrespective of whether systemic therapy was subsequently administered. Patients with adrenal pheochromocytoma were excluded.

### 2.3. Clinicopathological and Molecular Variables

Demographic and clinical data—age at diagnosis, sex, Eastern Cooperative Oncology Group (ECOG) performance status, comorbidities, presenting symptoms, and family history of a hereditary syndrome—were extracted from medical records and are summarized in Table 1. Functional status was defined by catecholamine secretion, based on plasma free metanephrine, normetanephrine, and dopamine together with 24 h urinary fractionated metanephrines and methoxytyramine. Histopathological evaluation comprised immunohistochemistry and the Grading of Adrenal Pheochromocytoma and Paraganglioma (GAPP) system, which integrates histological pattern, cellularity, comedo necrosis, capsular or vascular invasion, the Ki-67 labeling index, and catecholamine phenotype; tumors were graded as well (0–2), moderately (3–6), or poorly differentiated (7–10) [14]. The Ki-67 index was additionally analyzed as a continuous variable and dichotomized at 3% [15]. For continuous statistical evaluations, Ki-67 labeling index values reported as ‘<1%’ were numerically assigned a value of 0.5%. The 3% threshold was specified a priori from a published external cohort [15] and was not derived from, or optimized within, the present dataset; no data-driven cutoff search was performed. The neutrophil-to-lymphocyte ratio (NLR) was calculated from the baseline complete blood count. For the Cox models, NLR was dichotomized at the cohort median of 2.71. Molecular profiling, where tumor tissue and clinical indication allowed, was performed by tumor-based next-generation sequencing of tumor tissue, covering paraganglioma susceptibility and driver genes including *SDHB*, *SDHD*, *RET*, *NF1*, and *VHL*; molecular results were therefore available only in a subset of patients. Susceptibility-gene testing was performed by tumor-based next-generation sequencing in 8 patients; in 2 further patients, *SDHB* status was assessed by immunohistochemistry rather than by sequencing. Both are reported together as tumor-based testing (*n* = 10) and are distinguished in Table 2. Because sequencing was performed on tumor tissue without a paired normal sample, the variants detected cannot be shown to be exclusively somatic in origin. PD-L1 expression and mismatch repair (MMR) protein expression—the latter interpreted as proficient or deficient mismatch repair rather than as a molecular test of microsatellite instability—were evaluated by immunohistochemistry where performed. Because molecular profiling was restricted to tumor-only sequencing, germline pathogenic variants were not systematically assessed, and a hereditary syndrome was unknown in most patients.

### 2.4. Tumor Localization, Staging, and Imaging

Tumor localization was classified as parasympathetic head and neck (e.g., carotid body, jugulotympanic, vagal, or laryngeal) or sympathetic extra-adrenal (e.g., paravertebral thoracic, mediastinal, retroperitoneal/para-aortic, organ of Zuckerkandl, urinary bladder, or pelvic). Tumor size and laterality were documented. No patient had multifocal or synchronous paragangliomas, so tumor size and localization were assigned from the single primary lesion in every case. Two tumors with equivocal primary localization—a pretracheal nodal lesion and a duodenal tumor—were recorded as not classifiable. Stage at diagnosis was assigned according to the AJCC 8th edition staging system, which applies only to pheochromocytoma and sympathetic (extra-adrenal) paragangliomas. Because the AJCC 8th edition does not provide a staging system for head and neck paragangliomas, all parasympathetic tumors were recorded as not applicable for TNM staging; carotid body tumors were additionally classified by the Shamblin system. Functional and anatomical imaging comprised somatostatin-receptor ([68Ga]-DOTATATE) positron emission tomography (PET) and [18F]-fluorodeoxyglucose (FDG) PET maximum standardized uptake values (SUVmax), metaiodobenzylguanidine (MIBG) scintigraphy, and cross-sectional computed tomography (CT) or magnetic resonance imaging (MRI).

### 2.5. Treatment and Response Assessment

Treatment data captured surgery and resection-margin status, additional therapy, locoregional recurrence and re-resection, and the indication for systemic therapy (locally inoperable or metastatic disease). For patients who received systemic therapy, first- and second-line regimens, treatment dates, and outcomes were recorded; these data are summarized in Table 3. Treatment response was assessed by the treating investigator on cross-sectional imaging according to the Response Evaluation Criteria in Solid Tumors (RECIST) version 1.1; the best overall response (unconfirmed) was categorized as complete response (CR), partial response (PR), stable disease (SD), or progressive disease (PD).

**Table 2:** Tumor-based susceptibility-gene and SDHB immunohistochemical findings with corresponding outcomes (*n* = 10).

Pt	Tumor Finding	Localization (Site)	Size, mm	Ki-67, %	GAPP	Recurrence	Systemic Therapy	Status (FU, mo)
A	*SDHB* mutant	Parasympathetic (carotid body)	35	8	MD	No	No	Alive (22)
B	*SDHB* mutant	Parasympathetic (jugulotympanic)	36	9	MD	Yes	Yes	Dead (99)
C	*SDHB* mutant	Parasympathetic (jugulotympanic)	50	<1	MD	No	No	Alive (63)
D	*SDHB* mutant	Parasympathetic (vagal)	65	8	MD	Yes	Yes	Alive (9)
E	*SDHB* wild-type	Parasympathetic (carotid body)	60	3	WD	Yes	Yes	Alive (227)
F	*SDHB* expression retained (IHC)	Sympathetic (retroperitoneal)	47	2	MD	No	No	Alive (7)
G	*SDHB* expression retained (IHC)	Sympathetic (retroperitoneal)	51	<1	WD	No	No	Alive (10)
H	*NF1* mutant	Sympathetic (retroperitoneal)	96	31	MD	Yes	Yes	Dead (78)
I	*VHL* mutant	Sympathetic (urinary bladder)	24	1	WD	No	Yes	Alive (43)
J	*TP53* mutant	Sympathetic (paravertebral lumbar/sacral)	30	4	MD	No	No	Alive (116)

Patient letters are arbitrary and do not correspond to the numbering used in Table 4. Follow-up durations are rounded to the nearest month; the corresponding values in Table 4 are given to one decimal place. FU, follow-up; GAPP, Grading of Adrenal Pheochromocytoma and Paraganglioma; IHC, immunohistochemistry; MD, moderately differentiated; WD, well differentiated. One additional patient (retroperitoneal/para-aortic, stage IV) underwent PD-L1 and mismatch repair immunohistochemistry rather than susceptibility-gene testing; the tumor was PD-L1 positive (CPS, 5) with retained MMR protein expression (proficient mismatch repair).

**Table 3:** Disease course and treatment (*N* = 43).

Variable	Value
Surgery, yes/no	40 (93.0)/3 (7.0)
Surgical margin (resected, *n* = 40): R0/R1/R2	29 (72.5)/11 (27.5)/0
Additional therapy (resected, *n* = 40): none/radiotherapy/systemic	28 (70.0)/6 (15.0)/6 (15.0)
Recurrence (among resected, *n* = 40)	13 (32.5)
Number of recurrences (1/2)	10/3
Re-resection (among 13)	6 (46.2)
Metastatic, recurrent, or inoperable disease requiring systemic therapy/received systemic therapy	9 (20.9)/9 (20.9)
Vital status: deceased	5 (11.6)
First-line regimen (*n* = 9): PRRT/CVD/TMZ/sunitinib	3/3/2/1
Metastatic sites (*n* = 9) ^†^	bone 6, liver 5, lymph node 3, lung 2, peritoneal 2
Local ablative therapy: none/metastasectomy/palliative RT	5/2/2
First-line best response (evaluable *n* = 8): PD/SD/CR–PR	5/2/1
ORR/DCR	12.5% (0.3–52.7)/37.5% (8.5–75.5)
Median first-line PFS, months (95% CI) (PFS-estimable *n* = 9)	13.5 (5.0–31.0)
Second-line therapy	4 (sunitinib-based 3; cabozantinib 1)

Data are *n* (%) unless otherwise stated. ^†^ Multiple sites per patient. The number of recurrences refers to resected patients with recurrence. CVD, cyclophosphamide–vincristine–dacarbazine; DCR, disease control rate; ORR, objective response rate; PFS, progression-free survival; PRRT, peptide receptor radionuclide therapy; RT, radiotherapy; TMZ, temozolomide. ORR/DCR with Clopper–Pearson 95% CI.

### 2.6. Endpoints

The primary outcome was recurrence-free survival (RFS), analyzed in patients who underwent resection and defined as the interval from the date of surgery to the first locoregional or distant recurrence or death from any cause, whichever occurred first. Patients without an event were censored at the last disease assessment or at the data cutoff, whichever came first. Recurrences were counted once, at the first documented event; subsequent recurrences did not contribute additional events. Patients with metastatic disease documented at diagnosis who underwent resection of the primary tumor were included in the recurrence-free survival analysis, with the first subsequent locoregional or distant event, or death from any cause, counted as an event. Because the composite included death from any cause, a cause-specific sensitivity analysis censoring deaths unrelated to paraganglioma was performed (Section 2.7).

Secondary outcomes were overall survival (OS); the objective response rate (ORR), disease control rate (DCR), and first-line progression-free survival (PFS) among patients who received systemic therapy; and exploratory associations between clinicopathological or molecular features and recurrence or metastatic disease. OS was defined as the interval from diagnosis to death from any cause, with surviving patients censored at the date of last follow-up or the data cutoff; given the small number of deaths, OS was analyzed descriptively. First-line PFS was defined as the interval from initiation of first-line systemic therapy to documented progression or death from any cause. ORR was defined as the proportion of patients with a best response of complete or partial response, and DCR as the proportion with complete response, partial response, or stable disease. ORR and DCR were calculated among patients with at least one post-baseline tumor assessment; first-line PFS was calculated among all patients who started first-line therapy.

### 2.7. Statistical Analysis

Continuous variables were summarized as median (interquartile range, IQR) and categorical variables as frequencies and percentages. Time-to-event outcomes were estimated using the Kaplan–Meier method, with medians and 95% confidence intervals (CIs); the median follow-up duration was estimated using the reverse Kaplan–Meier method. Survival distributions were compared with the log-rank test, and prognostic associations were evaluated using Cox proportional-hazards models, with results expressed as hazard ratios (HRs) and 95% CIs. ORR and DCR were reported with exact (Clopper–Pearson) 95% CIs. Associations between categorical variables were examined with the chi-square or Fisher exact test, and continuous variables were compared between groups using the Mann–Whitney U or Kruskal–Wallis test, as appropriate. A two-sided *p* < 0.05 was considered statistically significant.

*Missing data.* No imputation was performed. Univariable Cox models were fitted on complete cases, so the effective sample size differed between models: all 40 resected patients contributed to the models for Ki-67, resection margin, tumor size, mitotic count, GAPP grade, and the neutrophil-to-lymphocyte ratio; 39 contributed to the model for tumor localization (1 patient with a tumor of equivocal localization excluded); and 23 patients with 9 events contributed to the model for functional status (17 patients of unknown status excluded). Hereditary syndrome status was not entered into any Cox model because it was unknown in 36 of 43 patients. The catecholamine component of the GAPP score was recorded from the pathological and biochemical source records and was available for all 43 tumors; it is distinct from the clinical functional classification, which was unknown in 17 patients, so a complete GAPP score could be assigned in every case. Patients with unknown values were not analyzed as a separate category and were not combined with a reference group.

*Multiple testing.* Eight prespecified univariable Cox models were fitted. Because multivariable adjustment was precluded by the limited number of events, the resulting *p* values were additionally adjusted for multiplicity using the Bonferroni method and Benjamini–Hochberg control of the false discovery rate; both adjusted values are reported alongside the unadjusted values for the covariates that were significant on unadjusted testing, and the remaining covariates are reported as not surviving adjustment.

*Proportional hazards and sensitivity analyses.* The proportional-hazards assumption was assessed using Schoenfeld residuals. Where it was violated, three complementary analyses that do not assume proportional hazards were performed. First, restricted mean survival time (RMST) was estimated for each Ki-67 group at horizons of 60 and 120 months, with 95% CIs and *p* values for the between-group difference obtained from 2000 bootstrap resamples (percentile method). Second, recurrence-free survival rates at the 24-, 60-, and 120-month landmarks were compared using a point-wise test of the difference in survival probability (that is, fixed–time-point comparisons of survival probability rather than analyses conditioned on being event-free at the landmark); the number of patients remaining at risk is reported where it constrains interpretation. Third, a piecewise Cox model with a time-by-covariate interaction was fitted, splitting follow-up at 36 months and reporting period-specific hazard ratios; a split at 24 months was not estimable because of complete separation.

*Exploratory adjustment and correlation.* Because the events-per-variable ratio precluded standard multivariable modeling, the four covariates significant on univariable analysis were additionally entered into penalized Cox models (ridge and LASSO, penalty 0.1) to examine mutual attenuation; these models are exploratory and are not presented as tests of independence. The penalty was fixed a priori rather than selected by cross-validation, and confidence intervals and *p* values obtained from penalized models are approximate and are reported descriptively. Correlations between the continuous Ki-67 index and the individual GAPP components and mitotic count were quantified with the Spearman rank coefficient.

*Subgroup analyses.* Because parasympathetic and sympathetic paragangliomas differ in natural history, the association between Ki-67 and recurrence-free survival was re-examined in resected sympathetic tumors alone and in a Cox model stratified by tumor localization. Subgroup analyses were not powered for formal inference and are reported descriptively.

Univariable Cox models were fitted for prespecified prognostic factors; given the limited number of events (events per variable < 10), no conventional unpenalized multivariable Cox model was fitted, and these analyses are considered exploratory. Analyses were performed using IBM SPSS Statistics, version 25 (IBM Corp., Armonk, NY, USA) and Python version 3.11 (lifelines version 0.30.3, statsmodels version 0.14.6, SciPy version 1.17.1, and NumPy version 2.4.6). The study is reported in accordance with the STROBE statement for observational studies.

## 3. Results

### 3.1. Patient and Tumor Characteristics

Forty-three patients with paraganglioma were included. The median age at diagnosis was 44 years (IQR, 33.0–55.5), and 26 patients (60.5%) were female. Performance status was ECOG 0–1 in 41 patients (95.3%). A hereditary syndrome or family history was documented in 1 patient (2.3%), absent in 6 (14.0%), and unknown in 36 (83.7%). Catecholamine secretion was functional in 13 patients (30.2%), non-functional in 13 (30.2%), and of unknown status in 17 (39.5%). Tumors were sympathetic (extra-adrenal) in 24 patients (55.8%) and parasympathetic (head and neck) in 17 (39.5%), and localization was not classifiable in 2 (4.7%).

Among the 24 sympathetic tumors, the primary site was retroperitoneal or para-aortic in 13 (54.2%), urinary bladder in 4 (16.7%), paravertebral thoracic in 2, organ of Zuckerkandl in 2, paravertebral lumbar or sacral in 2, and mediastinal in 1. Among the 17 parasympathetic tumors, the site was jugulotympanic in 9 (52.9%), carotid body in 6 (35.3%), vagal in 1, and other head and neck in 1. The two tumors of equivocal localization were a pretracheal nodal lesion and a duodenal tumor. No patient had multifocal or synchronous paragangliomas.

The median largest tumor diameter was 38.0 mm (IQR, 23.0–62.5). By GAPP grade, 14 tumors (32.6%) were well, 26 (60.5%) moderately, and 3 (7.0%) poorly differentiated; the median GAPP total score was 3 (IQR, 2–4) and the median Ki-67 index was 2% (IQR, 0.5–3).

Six patients (14.0%) had a second primary neoplasm: chronic lymphocytic leukemia, multiple myeloma, NF1-associated neurofibroma, gastrointestinal stromal tumor, bladder carcinoma, and HER2-positive breast carcinoma.

Baseline characteristics are summarized in Table 1.

### 3.2. Molecular and Immunohistochemical Findings

Tumor-based susceptibility-gene sequencing was performed in 8 patients and *SDHB* immunohistochemistry in 2 further patients, giving tumor-based information in 10 patients (23.3%). Among these, *SDHB* was mutant in four and wild-type in one, with retained SDHB expression by immunohistochemistry in two further tumors; *NF1*, *VHL*, and *TP53* alterations were each identified in one patient. Individual findings and corresponding outcomes are shown in Table 2. In one further patient, who did not undergo susceptibility-gene testing, PD-L1 expression (combined positive score [CPS], 5) and proficient mismatch repair with retained MMR protein expression were documented by immunohistochemistry.

All four *SDHB*-mutant tumors were parasympathetic (carotid body, one; jugulotympanic, two; vagal, one). Two of the four developed recurrence and received first-line systemic therapy, one of whom died at 99 months; the remaining two were recurrence-free at 22 and 63 months of follow-up.

Germline testing was not performed, so transcriptional cluster assignment and germline syndrome classification could not be determined.

### 3.3. Disease Course and Treatment

Forty patients (93.0%) underwent surgical resection; the margin was R0 in 29 (72.5%) and R1 in 11 (27.5%), with no R2 resections. Among the 40 resected patients, 12 (30.0%) received adjuvant therapy (radiotherapy in 6 and systemic therapy in 6), while 28 (70.0%) received none. Among resected patients, 13 (32.5%) developed locoregional or distant recurrence, of whom 6 (46.2%) underwent re-resection. Treatment details are presented in Table 3.

Separately, first-line systemic therapy for metastatic, recurrent, or inoperable disease was administered to nine patients (20.9%). Two did not undergo resection: one had locally inoperable disease without distant metastasis at diagnosis and received definitive radiotherapy to the primary tumor before developing metastatic disease, and one had metastatic disease at diagnosis. Of the seven patients who underwent resection, three had metastatic disease documented at diagnosis and underwent resection of the primary tumor, and four were free of metastatic disease at diagnosis and developed metastatic or recurrent disease during follow-up.

With respect to stage at diagnosis, three of these nine patients had AJCC stage IV sympathetic disease; two had sympathetic tumors staged II and III at diagnosis and developed metastatic disease during follow-up; and four had head and neck tumors, which the AJCC 8th edition does not stage and which are therefore recorded as not applicable in Table 1. One of these four head and neck tumors was documented as metastatic at diagnosis in the source records. The three patients recorded as stage IV in Table 1 therefore correspond to the sympathetic tumors only.

First-line systemic regimens were [177Lu]-based peptide receptor radionuclide therapy in three patients, CVD-based chemotherapy in three, temozolomide in two, and sunitinib in one; metastasectomy and palliative radiotherapy were used as local ablative measures in two patients each. Individual clinicopathological features, treatment, and outcomes for these nine patients are detailed in Table 4, and best response by regimen in Supplementary Table S1.

**Table 4:** Individual characteristics and outcomes of the nine patients who received first-line systemic therapy.

Pt	Age/Sex	Localization (Site)	Stage	Surgery (Margin)	Size, mm	Ki-67, %	GAPP	Sites of Active Disease	First-Line Regimen	Best Response	**PFS, mo**	**Local Ablative**	**Second Line**	**Status (FU, mo)**
1	23/F	Parasympathetic (carotid body)	NA ^†^	Yes (R1)	60	3	WD	Local, liver, bone, LN	[177Lu]-DOTATATE	NE	16.6+	Palliative RT	—	Alive (226.8)
2	32/M	Parasympathetic (jugulotympanic)	NA	No	31	90 ^#^	PD	Local, bone, LN	CVD	PD	5.0	— ^¶^	—	Dead (13.3)
3	66/M	Parasympathetic (jugulotympanic)	NA	Yes (R1)	36	9	MD	Bone	[177Lu]-DOTATATE + zoledronate	PD	10.1	Palliative RT	—	Dead (99.1)
4	30/F	Parasympathetic (vagal)	Metastatic (not staged) ^‡^	Yes (R1)	65	8	MD	Local, lung, bone	Sunitinib	SD	4.6+ ^§^	—	—	Alive (8.6)
5	58/F	Sympathetic (paravertebral thoracic)	III	Yes (R1)	75	5	MD	Liver	Temozolomide	SD	31.0	Metastasectomy	—	Dead (65.6)
6	50/F	Sympathetic (retroperitoneal/para-aortic)	II	Yes (R0)	80	6	PD	Liver, peritoneum (omentum, ovary)	[177Lu]-DOTATATE + octreotide	PD	15.4	—	Octreotide + sunitinib	Alive (120.9)
7	46/F	Sympathetic (retroperitoneal/para-aortic)	IV	No	140	4	MD	Local, lung, liver, bone, LN, peritoneum	CVD + zoledronate	PD	11.7	—	Sunitinib	Alive (31.7)
8	25/M	Sympathetic (retroperitoneal/para-aortic)	IV	Yes (R1)	96	31	MD	Liver	CVD	PD	6.6	Metastasectomy	Sunitinib	Dead (78.4)
9	56/F	Sympathetic (urinary bladder)	IV	Yes (R0)	24	1	WD	Bone	Temozolomide	PR	13.5	—	Cabozantinib	Alive (43.4)

Patient numbers are arbitrary and do not correspond to the lettering used in Table 2. ^†^ Head and neck tumors are not staged by the AJCC 8th edition; this carotid body tumor was Shamblin III. ^‡^ Recorded as stage IV in the source records; classified as “not applicable” in Table 1 because the AJCC 8th edition does not stage head and neck tumors. ^#^ The Ki-67 index of 90.0% in Patient 2 was pathologically confirmed from biopsy tissue, reflecting a highly aggressive, poorly differentiated course. ^§^ The surgical-bed lesion is included among the sites of disease present at the start of first-line therapy; it and the metastatic sites remained stable on sunitinib, so no progression event occurred and first-line progression-free survival is censored at the data cutoff. ^¶^ Patient 2 did not undergo resection; definitive radiotherapy was delivered to the primary tumor, and metastatic disease developed subsequently. The local ablative therapy column records treatment directed at metastatic sites only. + Censored. CVD, cyclophosphamide–vincristine–dacarbazine; FU, follow-up; GAPP, Grading of Adrenal Pheochromocytoma and Paraganglioma; LN, lymph node; MD, moderately differentiated; NA, not applicable; NE, not evaluable; PD (response), progressive disease; PD (GAPP), poorly differentiated; PR, partial response; RT, radiotherapy; SD, stable disease; WD, well differentiated.

One of the nine patients was not evaluable for response because no post-baseline tumor assessment was available. Among the eight patients evaluable for first-line response, the ORR was 12.5% (1/8; 95% CI, 0.3–52.7) and the DCR was 37.5% (3/8; 95% CI, 8.5–75.5). First-line progression-free survival was estimable in all nine patients, the patient without a post-baseline tumor assessment contributing a censored observation rather than a progression event; the median was 13.5 months (95% CI, 5.0–31.0). Four patients received second-line therapy (sunitinib-based in three and cabozantinib in one).

### 3.4. Survival Outcomes

**Figure 1:**
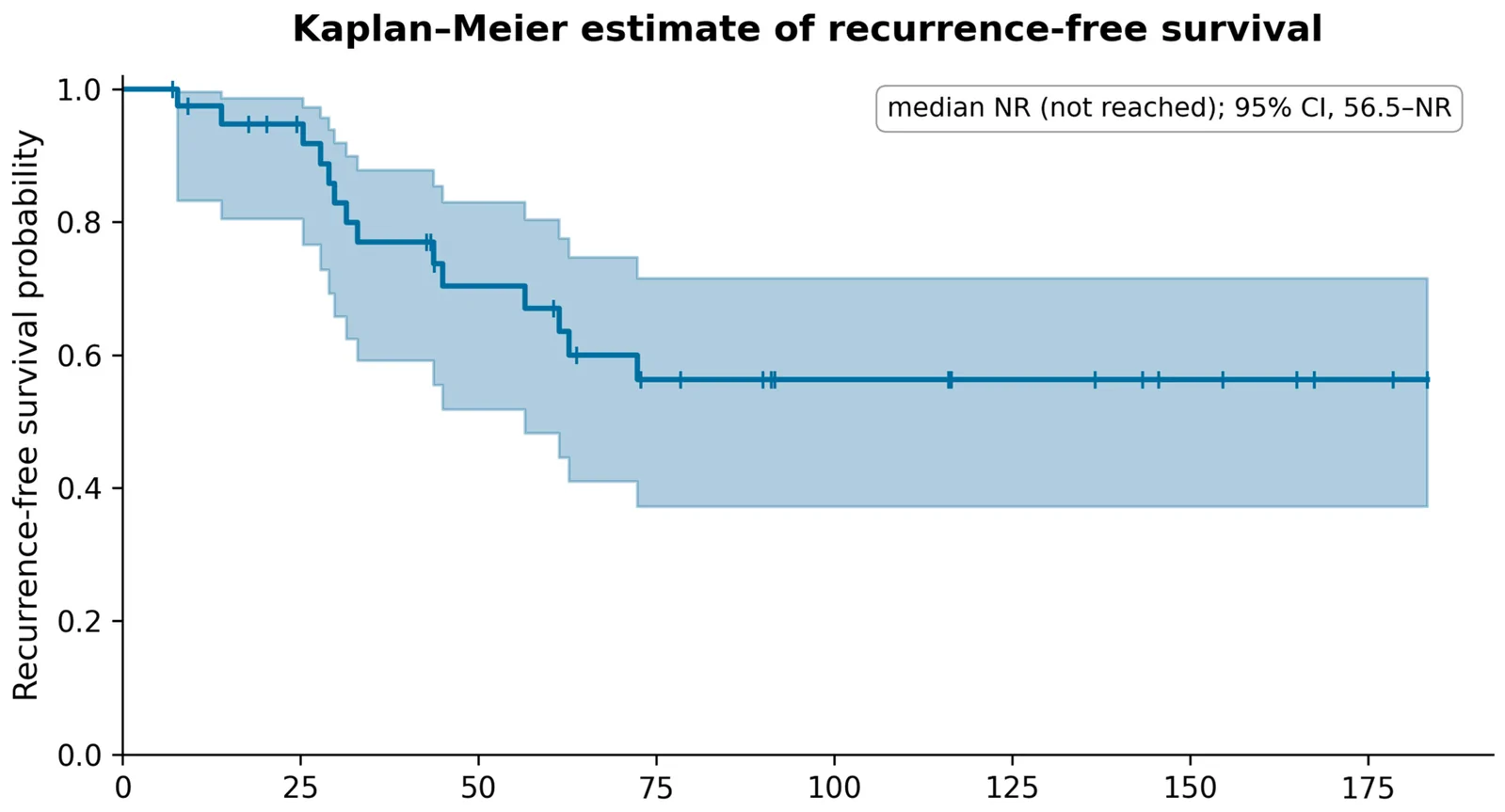
Kaplan–Meier estimate of recurrence-free survival in the resected cohort (*n* = 40). The median was not reached; tick marks denote censored observations and the shaded area the 95% confidence interval. NR, not reached.

**Figure 2:**
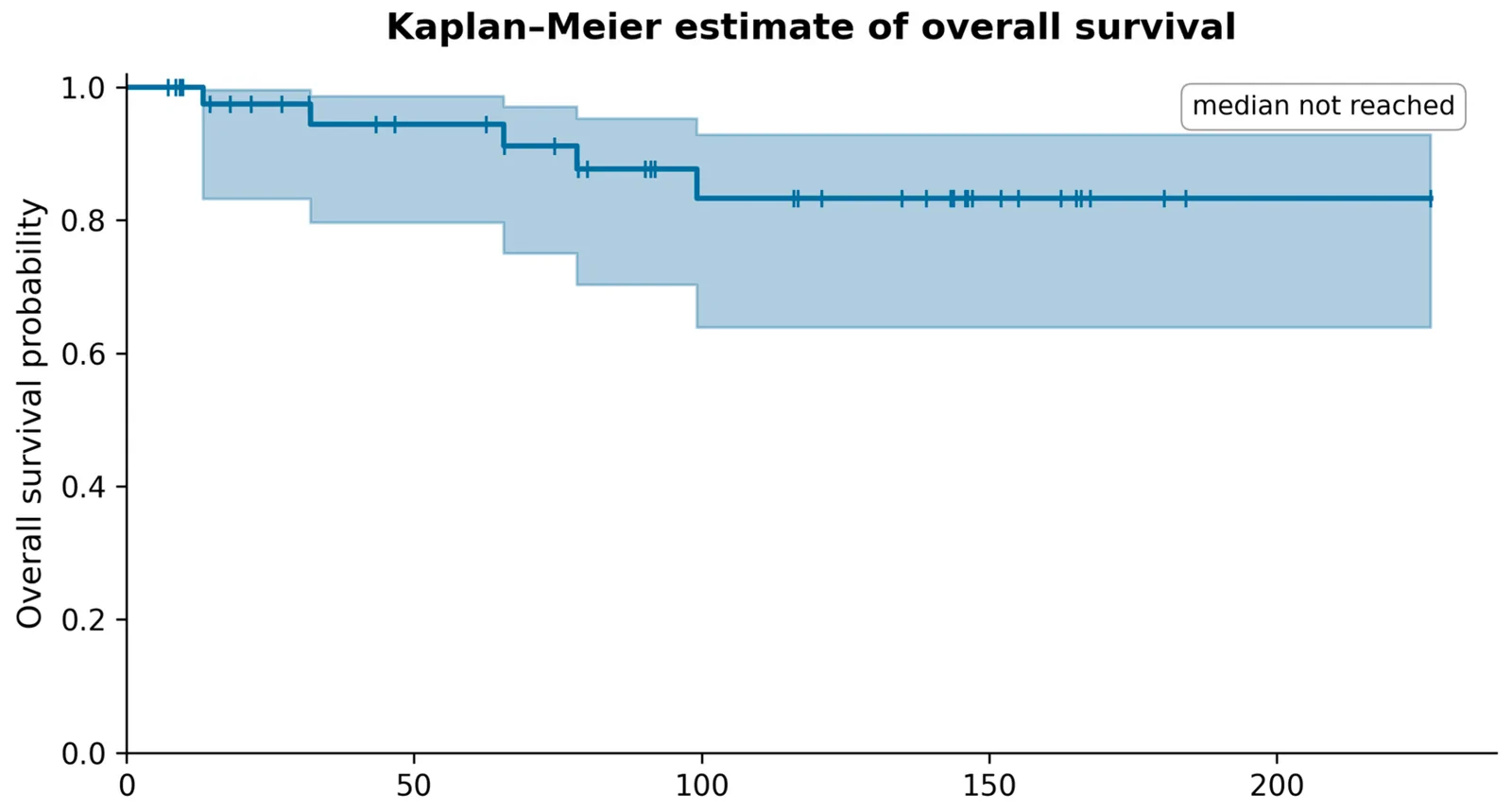
Kaplan–Meier estimate of overall survival in the whole cohort (*n* = 43). The median was not reached; the shaded area denotes the 95% confidence interval.

After a median follow-up of 116.8 months (95% CI, 74.4–143.8), 5 of 43 patients (11.6%) had died. The primary endpoint, recurrence-free survival, defined as the interval from resection to the first locoregional or distant recurrence or death from any cause, was analyzed in the 40 resected patients, among whom 14 events occurred: 13 first recurrences and 1 death without documented recurrence. This single death was unrelated to paraganglioma; a cause-specific sensitivity analysis is reported in Section 3.6. Median recurrence-free survival was not reached (95% CI, 56.5 months—not reached); recurrence-free survival was 97.4% (95% CI, 83.2–99.6) at 12 months, 94.7% (95% CI, 80.5–98.7) at 24 months, and 67.1% (95% CI, 48.2–80.3) at 60 months (Figure 1). For overall survival, median survival was not reached, with rates of 94.5% (95% CI, 79.6–98.6) at 60 months and 83.3% (95% CI, 63.8–92.9) at 120 months (Figure 2); given the small number of deaths, overall survival is reported descriptively.

### 3.5. Prognostic Factors for Recurrence-Free Survival

**Figure 3:**
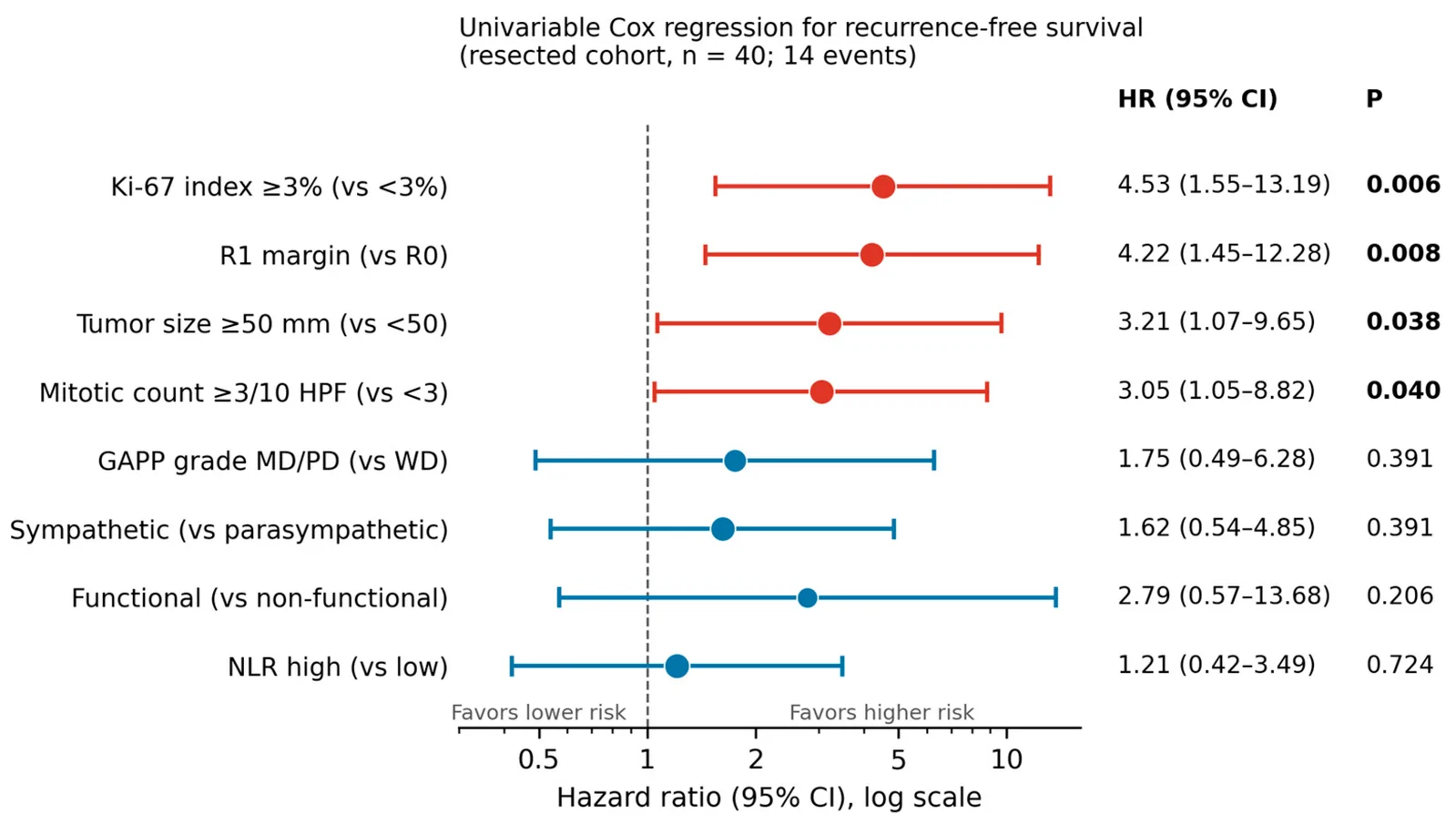
Univariable Cox regression for recurrence-free survival in the resected cohort (*n* = 40; 14 events). Model-specific sample sizes were *n* = 39 for tumor localization and *n* = 23 (9 events) for functional status; all other models included all 40 resected patients. Circles denote hazard ratios and horizontal lines the 95% confidence intervals (log scale). Red circles and bold *p* values indicate *p* < 0.05 on unadjusted testing; blue circles indicate *p* ≥ 0.05. CI, confidence interval; HPF, high-power field; HR, hazard ratio; NLR, neutrophil-to-lymphocyte ratio.

In exploratory univariable Cox regression among resected patients, a Ki-67 index ≥ 3% (HR, 4.53; 95% CI, 1.55–13.19; *p* = 0.006) and an R1 resection margin (HR, 4.22; 95% CI, 1.45–12.28; *p* = 0.008) were associated with shorter recurrence-free survival, while tumor size ≥ 50 mm (HR, 3.21; 95% CI, 1.07–9.65; *p* = 0.038) and a mitotic count ≥ 3/10 HPF (HR, 3.05; 95% CI, 1.05–8.82; *p* = 0.040) showed nominally significant associations. GAPP grade, tumor localization, functional status, and the neutrophil-to-lymphocyte ratio were not significantly associated with recurrence-free survival (Figure 3).

All models included the 40 resected patients except those for tumor localization (*n* = 39; 1 patient with a tumor of equivocal localization excluded) and functional status (*n* = 23, 9 events; 17 patients of unknown status excluded), which were fitted on complete cases.

Analyzed as a continuous variable, the Ki-67 index was associated with recurrence-free survival per 1% absolute increase (HR, 1.05; 95% CI, 1.01–1.09; *p* = 0.012), per 5% increase (HR, 1.28; 95% CI, 1.06–1.55; *p* = 0.012), and per doubling (HR, 1.39; 95% CI, 1.10–1.76; *p* = 0.006).

Because eight univariable models were fitted, *p* values were additionally adjusted for multiple testing. A Ki-67 index ≥ 3% remained significant after Bonferroni correction (adjusted *p* = 0.048) and after Benjamini–Hochberg control of the false discovery rate (adjusted *p* = 0.032), as did an R1 margin under false discovery rate control (adjusted *p* = 0.032; Bonferroni *p* = 0.064). Tumor size ≥ 50 mm and mitotic count ≥ 3/10 HPF did not remain significant after adjustment (false discovery rate-adjusted *p* = 0.080 for both).

**Figure 4:**
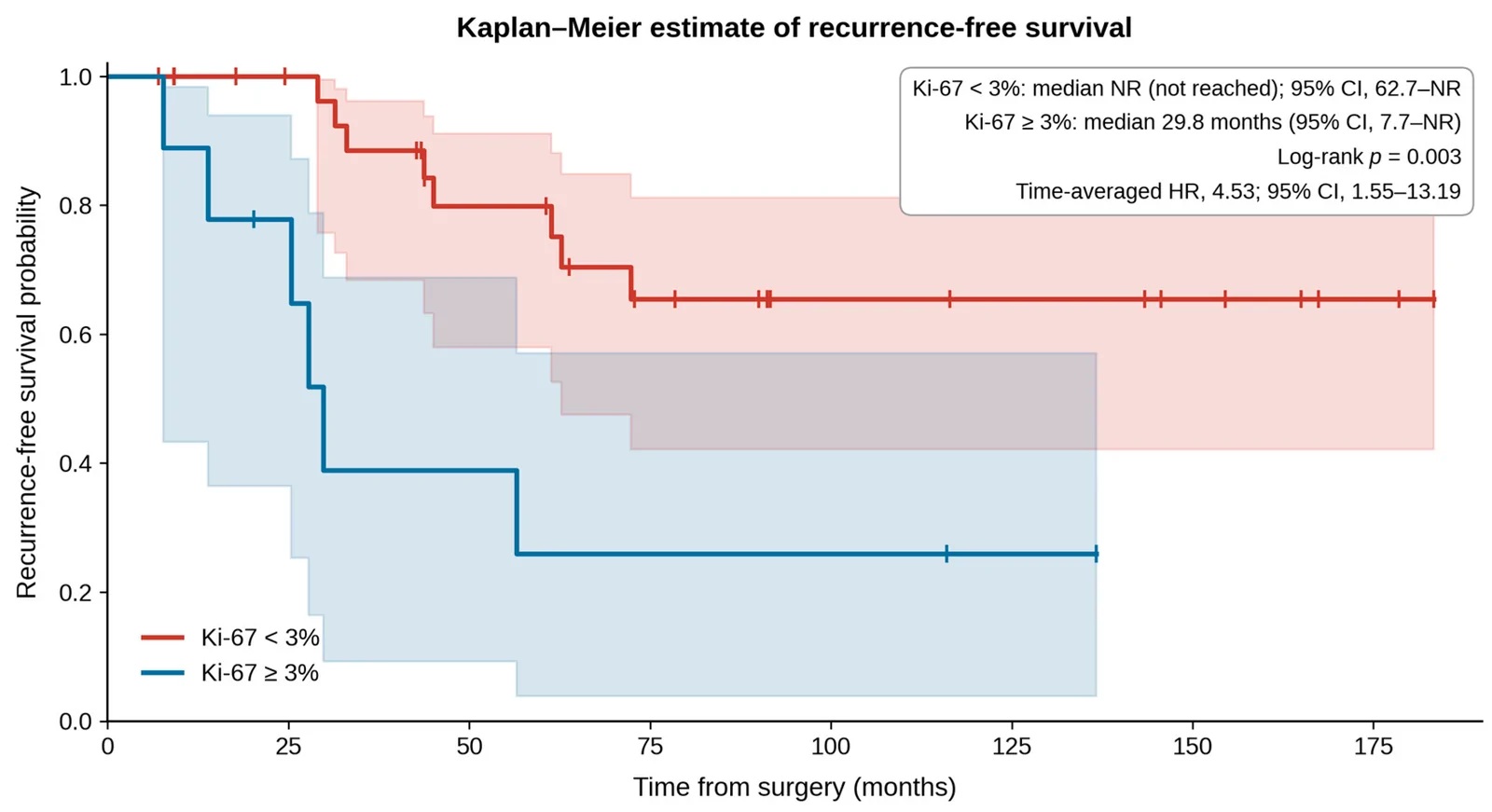
Kaplan–Meier estimates of recurrence-free survival according to the Ki-67 index (<3% versus ≥3%) in the resected cohort (*n* = 40); log-rank *p* = 0.003. Tick marks denote censored observations and the shaded areas the 95% confidence intervals. CI, confidence interval; HR, hazard ratio; NR, not reached.

By the Ki-67 threshold, median recurrence-free survival was 29.8 months (95% CI, 7.7–not reached) for patients with a Ki-67 index ≥ 3% (*n* = 9) versus not reached for those with an index < 3% (*n* = 31) (log-rank *p* = 0.003), with 60-month recurrence-free survival rates of 25.9% and 79.8%, respectively (Figure 4). No conventional unpenalized multivariable Cox model was fitted because of the limited number of events (events per variable < 10), and the proportional-hazards assumption was violated for Ki-67 and tumor size; the corresponding hazard ratios therefore represent time-averaged effects, and sensitivity analyses addressing this, together with exploratory penalized models, are presented in Section 3.6.

### 3.6. Sensitivity and Subgroup Analyses

Because the proportional-hazards assumption was violated for Ki-67, the association with recurrence-free survival was re-examined using methods that do not assume proportional hazards. Restricted mean survival time up to 60 months was 55.3 months (95% CI, 51.3–58.9) for a Ki-67 index < 3% and 36.0 months (95% CI, 22.7–49.7) for an index ≥ 3%, a difference of 19.3 months (95% CI, 5.7–32.8; bootstrap *p* = 0.006). Up to 120 months, the corresponding values were 95.4 months (95% CI, 80.7–108.6) and 51.6 months (95% CI, 23.9–81.4), a difference of 43.8 months (95% CI, 9.7–74.7; bootstrap *p* = 0.016).

Landmark recurrence-free survival rates for a Ki-67 index < 3% versus ≥3% were 100% versus 77.8% (95% CI, 36.5–93.9) at 24 months, 79.8% (95% CI, 58.0–91.1) versus 25.9% (95% CI, 3.9–57.0) at 60 months (*p* = 0.005), and 65.4% (95% CI, 42.2–81.2) versus 25.9% (95% CI, 3.9–57.0) at 120 months (*p* = 0.045). No event occurred in the low–Ki-67 group by 24 months, so the 24-month comparison is reported descriptively; only one patient with a Ki-67 index ≥ 3% remained at risk at 120 months.

In a piecewise Cox model splitting follow-up at 36 months, the hazard ratio for a Ki-67 index ≥ 3% was 9.11 (95% CI, 2.14–38.80; *p* = 0.003) during the first 36 months and 1.41 (95% CI, 0.16–12.10; *p* = 0.75) thereafter (interaction *p* = 0.158). A model split at 24 months was not estimable because both events occurring before 24 months arose in the Ki-67 ≥ 3% group.

In a penalized (ridge) Cox model including Ki-67 ≥ 3%, R1 margin, tumor size ≥ 50 mm, and mitotic count, only a Ki-67 index ≥ 3% retained significance (HR, 3.45; 95% CI, 1.08–11.01; *p* = 0.037); under LASSO penalization all coefficients were shrunk toward zero and no covariate was retained in the model at the prespecified penalty. Spearman correlations between the continuous Ki-67 index and the non-proliferative GAPP components were weak (histological pattern, ρ = 0.07; capsular or vascular invasion, ρ = 0.13; comedo necrosis, ρ = 0.25; catecholamine type, ρ = −0.27; cellularity, ρ = 0.31), whereas correlations with mitotic count (ρ = 0.53) and the GAPP total score (ρ = 0.49) were moderate (Table S2).

In a cause-specific analysis censoring the single non-paraganglioma-related death rather than counting it as an event (13 events), the 60-month recurrence-free survival rate was 70.1%, and the association with a Ki-67 index ≥ 3% was attenuated to borderline significance (HR, 3.07; 95% CI, 1.00–9.43; *p* = 0.050). This death occurred in a patient with a Ki-67 index ≥ 3%; that stratum comprised only nine patients, so the attenuation reflects the removal of a single event from a small group.

Restricted to resected sympathetic tumors (*n* = 23; nine events; five with a Ki-67 index ≥ 3%), a Ki-67 index ≥ 3% remained associated with shorter recurrence-free survival (HR, 5.46; 95% CI, 1.42–21.02; *p* = 0.014; log-rank *p* = 0.006; Figure S1). In a Cox model stratified by tumor localization (*n* = 39; 14 events), the corresponding hazard ratio was 4.38 (95% CI, 1.48–12.94; *p* = 0.008). Among the 16 resected parasympathetic tumors, 5 events occurred; the median Ki-67 index was 2%, the median largest diameter 30 mm, and 5 resections were R1.

## 4. Discussion

In this single-center medical oncology cohort of 43 patients with paraganglioma followed for a median of 116.8 months, the disease was predominantly localized and surgically managed, recurrence-free survival was favorable overall, and overall survival was high. Among resected patients, a Ki-67 index ≥ 3% was associated with recurrence in exploratory univariable analysis, alongside an R1 resection margin and, nominally, larger tumor size and a higher mitotic count, whereas systemic therapy for metastatic disease yielded limited objective responses. Because 40 of 43 patients underwent resection and only 9 received systemic therapy, this cohort informs postoperative recurrence risk more than it evaluates medical oncology interventions, and we frame it accordingly. Its contribution lies in the duration of follow-up—a median of 116.8 months, sufficient to capture the late recurrences characteristic of these tumors—and in reporting postoperative outcomes and systemic-treatment experience within a single medical oncology referral setting, a perspective less often represented than surgical or endocrine series.

A central observation is that the Ki-67 index, analyzed at the 3% threshold, was associated with recurrence-free survival, while the composite GAPP grade was not. This contrast should not be over-read. Only three tumors were classified as poorly differentiated, so the comparison between GAPP grade and Ki-67 was underpowered, and the absence of an association for GAPP is at least as consistent with limited statistical power as with a genuine difference in prognostic value. We therefore refrain from concluding that the proliferative component accounts for the prognostic signal of GAPP; the observation that Ki-67 correlated only weakly with the non-proliferative GAPP components in our cohort is hypothesis-generating rather than confirmatory. In an independent validation of five scoring systems in 185 PPGLs, GAPP showed low discriminative ability (C-index, 0.547), lower than PASS (0.600) and substantially lower than COPPS (0.740) and ASES (0.706) [16]. The absence of an association between GAPP grade and recurrence in our cohort is therefore consistent with the limited discrimination reported in independent series, although our comparison was additionally underpowered. Ki-67 is the only objective, continuous parameter within GAPP [14], and recent data support a 3% cutoff for predicting progression, relapse, or metastasis [15]. The proportional-hazards assumption was violated for Ki-67, so the reported hazard ratio is a time-averaged summary rather than a constant effect. Point estimates were markedly higher during the first 36 months than thereafter, but the formal time-by-covariate interaction was not significant and the two interval estimates overlapped widely, so the temporal pattern should be read as hypothesis-generating rather than established. Analyses that do not require proportional hazards were concordant in direction—restricted mean recurrence-free survival was shorter by 19.3 months at 60 months and by 43.8 months at 120 months in the high-Ki-67 group. If confirmed, this time course would be clinically coherent, with proliferative activity predicting early rather than late recurrence and arguing for intensified surveillance in the initial postoperative years rather than a uniformly elevated long-term hazard; because the interaction test did not reach significance, we present it as a hypothesis for larger cohorts. Because only 9 resected patients had a Ki-67 index ≥ 3% and 14 events occurred overall, and because no covariate was retained under LASSO penalization, we do not claim that Ki-67 is an independent predictor or that it outperforms other risk factors; the association survived correction for multiple testing but remains exploratory. Because the current World Health Organization framework regards all paragangliomas as having metastatic potential and no single histological feature reliably separates indolent from aggressive tumors [10,13], objective proliferative markers such as Ki-67 are attractive adjuncts for risk stratification.

The association of an R1 margin and a tumor size ≥ 50 mm with shorter recurrence-free survival is biologically coherent and aligns with larger size being recognized as an adverse prognostic feature, together with a dopaminergic phenotype and incomplete resection [2,17]. The association with tumor size, like that with Ki-67, violated the proportional-hazards assumption and did not persist after correction for multiple testing, and should be regarded as nominal. Recurrences in our cohort occurred over a prolonged interval, consistent with reports that head and neck paragangliomas may recur many years after treatment and with guideline recommendations for long-term—often lifelong—surveillance [10,11,24]. In the metastatic setting, resection of the primary tumor has been associated with longer overall survival [25], underscoring the value of complete surgical control and of sustained follow-up, particularly for larger or margin-positive tumors.

Tumor localization (sympathetic versus parasympathetic) was not significantly associated with recurrence-free survival, although the cohort was small and such comparisons were underpowered. Because the two entities differ in natural history, we repeated the analysis within resected sympathetic tumors alone and in a model stratified by localization; the Ki-67 association persisted in both, indicating that it was not generated by pooling biologically distinct tumors. Notably, the parasympathetic tumors in this series did not behave indolently: four of the nine patients who required systemic therapy for metastatic or inoperable disease had head and neck primaries, and all four tumors with a tumor-detected *SDHB* mutation were parasympathetic. Two of these four *SDHB*-mutant patients developed recurrence and received systemic therapy, and one died. Although the numbers are small and testing was performed on tumor tissue without matched normal samples, this pattern is a reminder that head and neck location does not by itself indicate low risk. In the broader literature, extra-adrenal location and *SDHB*-related disease confer the highest metastatic risk [11]. An important caveat is that molecular profiling in our cohort was restricted to tumor-only next-generation sequencing in a minority of patients; germline testing was not performed, and a hereditary syndrome was therefore unknown in most patients. Consequently, cluster-based prognostication and the contribution of germline *SDHx* variants [5,6,7,8] could not be assessed, an acknowledged gap given their established relevance.

This gap bounds what our findings can support. With hereditary status unknown in 83.7% of patients, we cannot exclude that germline genotype confounds the observed associations, and a Ki-67-based risk estimate is not a substitute for genetic evaluation. Current guidance recommends genetic testing in all patients with paraganglioma [6], and nothing in these data alters that recommendation: Ki-67 stratification should be understood as complementary to, and never as a replacement for, genetic counseling and germline testing. The appropriate reading of our findings is narrower—that in settings where germline testing is not readily accessible, an inexpensive and universally reported pathological parameter carries prognostic information that can inform surveillance intensity while genetic evaluation is arranged. Whether Ki-67 retains prognostic value after adjustment for germline genotype is the question that molecularly annotated cohorts will need to answer.

In the small metastatic subgroup, first-line systemic therapy—comprising [177Lu]-based PRRT, CVD-based chemotherapy, temozolomide, and sunitinib—produced an objective response in a single patient and disease control in roughly one-third, with a median first-line progression-free survival of 13.5 months and a few patients proceeding to second-line treatment. These figures pool biologically distinct modalities and should be read as a description of one center’s experience rather than as comparative efficacy data. With no more than three patients per regimen, no regimen-specific conclusion can be drawn, and the individual responses reported in Supplementary Table S1 are provided for transparency rather than for comparison. The single objective response occurred with temozolomide, but a denominator of two precludes any inference about relative activity. These results are consistent with the modest, predominantly disease-stabilizing activity reported across retrospective systemic-therapy series [19,21,23] and with the favorable short-term stabilization observed in therapy-naive patients [22], as well as with the generally unfavorable prognosis of metastatic disease [12,18]. From a medical oncology perspective, they underscore the limited options for advanced paraganglioma and the need for genotype-informed, cluster-oriented strategies.

Two features of the cohort’s composition warrant comment. First, patients were accrued between June 2007 and September 2025, a period over which the diagnosis and management of these tumors changed substantially: somatostatin-receptor PET imaging progressively replaced or supplemented MIBG scintigraphy, the 2022 World Health Organization classification reframed all paragangliomas as tumors with metastatic potential, surveillance became more intensive and more protracted, and PRRT entered routine practice. Patients diagnosed later were therefore more likely to have small or asymptomatic recurrences detected, and more likely to receive systemic options unavailable to those treated earlier. Such era effects act in opposing directions on the observed recurrence rate and cannot be disentangled in a cohort of this size, but they temper any comparison with historical series. Second, because all patients were referred to a medical oncology department, the cohort is enriched for aggressive disease relative to unselected paraganglioma populations, and the recurrence and metastasis frequencies reported here should not be read as population estimates.

This study has several limitations. Its retrospective, single-center design and modest sample size limit statistical power; the number of recurrence events precluded multivariable modeling (events per variable < 10), so the prognostic analyses are exploratory and require cautious interpretation. Eight univariable models were tested; although the Ki-67 association persisted after Bonferroni and false-discovery-rate correction, the associations with tumor size and mitotic count did not, and should be regarded as nominal. Referral to a medical oncology unit likely enriched the cohort for aggressive or metastatic disease, so the recurrence and metastasis frequencies may overestimate those of unselected paraganglioma populations. The proportional-hazards assumption was violated for Ki-67 and tumor size, such that their hazard ratios reflect time-averaged effects. The primary endpoint was a composite of recurrence and death from any cause. One of the 14 events was a death unrelated to paraganglioma; when this event was censored in a cause-specific sensitivity analysis, the association between a Ki-67 index ≥ 3% and recurrence-free survival was attenuated to borderline significance (HR, 3.07; 95% CI, 1.00–9.43; *p* = 0.050). The central finding is therefore sensitive to the handling of a single event, which is an inherent consequence of the small number of events and should be weighed when interpreting the effect size. Six patients had a second primary neoplasm, a further reason to interpret all-cause composite endpoints cautiously in a cohort of this size. A related caveat concerns the composition of the analysis population: three resected patients had metastatic disease documented at diagnosis, so that a subsequent event in these patients represents progression of established disease rather than recurrence after complete resection. They were retained under the prespecified definition in Section 2.6, but their inclusion means that the recurrence-free survival estimates combine two clinically distinct processes, and the effect size should be read with that in mind. A formal sensitivity analysis restricted to patients without metastatic disease at diagnosis would be informative and is a priority for validation in larger cohorts. Overall survival was immature, with only five deaths, and is reported descriptively. The absence of germline testing, incomplete molecular and staging data, and reliance on some free-text variables further constrain interpretation. Response assessment was performed by the treating investigator without independent central review, and best responses were unconfirmed, which may overestimate response duration. Subgroup and sensitivity analyses were not powered for formal inference; in particular, only 1 patient with a Ki-67 index ≥ 3% remained at risk at 120 months, so the 10-year landmark comparison rests on very few observations. Strengths include a long and relatively complete follow-up, uniform management within a single medical oncology unit, and granular treatment and response data.

## 5. Conclusions

In this medical oncology cohort, paraganglioma was predominantly localized and carried a favorable long-term prognosis, yet recurrences accrued over many years. In exploratory univariable analysis, a Ki-67 index ≥ 3% was associated with shorter recurrence-free survival after resection (time-averaged HR, 4.53; 95% CI, 1.55–13.19), corresponding to a 19.3-month reduction in restricted mean recurrence-free survival at 60 months, with point estimates highest in the first 3 postoperative years, although the time-by-covariate interaction did not reach statistical significance. The association was not adjusted for other risk factors and does not establish independence from them, while systemic therapy for metastatic disease achieved limited objective responses in the small response-evaluable subgroup. These exploratory findings may support consideration of the Ki-67 index as an adjunct within risk-adapted, long-term surveillance in settings where germline testing is not readily available, and they do not displace guideline-recommended genetic evaluation. They warrant validation in larger, prospective, molecularly annotated cohorts.

## Figures and Tables

**Table 1:** Baseline characteristics of the paraganglioma cohort (*N* = 43).

Characteristic	Overall (*N* = 43)
Age at diagnosis, years—median (IQR)	44.0 (33.0–55.5)
Sex, female/male	26 (60.5)/17 (39.5)
ECOG 0–1/2/3	41 (95.3)/1 (2.3)/1 (2.3)
Hereditary syndrome: yes/no/unknown	1 (2.3)/6 (14.0)/36 (83.7)
Catecholamine: functional/non-functional/unknown	13 (30.2)/13 (30.2)/17 (39.5)
Localization: sympathetic/parasympathetic/not classifiable	24 (55.8)/17 (39.5)/2 (4.7)
Sympathetic site: retroperitoneal or para-aortic/urinary bladder/paravertebral thoracic/Zuckerkandl/paravertebral lumbar or sacral/mediastinal	13 (54.2)/4 (16.7)/2 (8.3)/2 (8.3)/2 (8.3)/1 (4.2)
Parasympathetic site: jugulotympanic/carotid body/vagal/other head and neck	9 (52.9)/6 (35.3)/1 (5.9)/1 (5.9)
Stage I/II/III/IV/not applicable/missing	5 (11.6)/11 (25.6)/5 (11.6)/3 (7.0)/17 (39.5)/2 (4.7)
Largest tumor diameter, mm—median (IQR)	38.0 (23.0–62.5)
GAPP grade: WD/MD/PD	14 (32.6)/26 (60.5)/3 (7.0)
GAPP total score—median (IQR)	3.0 (2.0–4.0)
Ki-67 index, %—median (IQR)	2 (0.5–3)
Mitotic count ≥ 3/10 HPF—*n* (%)	15 (34.9)
NLR—median (IQR)	2.71 (1.93–3.88)
Second primary neoplasm—*n* (%)	6 (14.0)

Data are *n* (%) unless otherwise stated. ECOG, Eastern Cooperative Oncology Group; GAPP, Grading of Adrenal Pheochromocytoma and Paraganglioma; HPF, high-power field; IQR, interquartile range; NLR, neutrophil-to-lymphocyte ratio; WD/MD/PD, well/moderately/poorly differentiated. The AJCC 8th edition TNM system applies only to sympathetic (extra-adrenal) paragangliomas, which account for all numeric stages (I–IV). The “not applicable” category denotes the 17 head and neck (parasympathetic) tumors, which the AJCC 8th edition does not stage and among which carotid body tumors were additionally graded by the Shamblin system; the “missing” category denotes the two tumors of equivocal localization (a pretracheal nodal lesion and a duodenal tumor), for which stage could not be assigned. These two categories are distinct and are not pooled.

## Data Availability

The data presented in this study are available on request from the corresponding author. The data are not publicly available owing to privacy and ethical restrictions.

## Supplementary Materials

The following supporting information can be downloaded at: https://www.mdpi.com/article/10.3390/diagnostics16162516/s1, Table S1: First-line best response and progression-free survival by regimen (*n* = 9 treated). Table S2: Spearman rank correlation coefficients between the Ki-67 index and the individual GAPP components. Figure S1: Kaplan–Meier estimates of recurrence-free survival according to the Ki-67 index, restricted to the 23 resected sympathetic (extra-adrenal) paragangliomas (9 events).
