# Supplementary material for: Clinicopathological Features, Tumor Localization and Treatment Outcomes in Paraganglioma: A Single-Center Medical Oncology Cohort
Source: Diagnostics (Basel). 2026 Aug 10;16(16):2516. doi: 10.3390/diagnostics16162516 (PMC13512060; doi:10.3390/diagnostics16162516)
Supplement: Supplementary file 1 [file diagnostics-16-02516-s001.zip › diagnostics-4435933-supplementary.pdf]

Supplementary Materials

Clinicopathological Features, Tumor Localization and Treatment Outcomes in Paraganglioma: A Single-Center Medical Oncology Cohort

Hatice Asoglu, Esra Asarkaya, Abdurrahman Aykut, Gunes Dorukhan Cavusoglu, Yasemin Aydınalp Camadan, Irem Kolsuz Turker, Hacer Demirkose, Tolga Koseci, Ertugrul Bayram, Gamze Akkus, Ramazan Asoglu, Seyda Erdogan, Ismail Oguz Kara

Table S1. First-line best response and progression-free survival by regimen (n = 9 treated).

Regimen	Treated	Response-evaluable	PD	SD	PR/CR	Individual PFS, months
[177Lu]-based PRRT	3	2	2	0	0	16.6+, 10.1, 15.4
CVD-based chemotherapy	3	3	3	0	0	5.0, 11.7, 6.6
Temozolomide	2	2	0	1	1	31.0, 13.5
Sunitinib	1	1	0	1	0	4.6+
**Total**	**9**	**8**	**5**	**2**	**1**	**Median 13.5 (95% CI, 5.0–31.0)**

Median progression-free survival was not calculated within regimens because no more than 3 patients received any single regimen; individual values are listed instead. Response was evaluable in 8 of the 9 treated patients; one patient treated with [177Lu]-based PRRT had no post-baseline tumor assessment. Progression-free survival was estimable in all 9 patients. Regimen-specific conclusions cannot be drawn from these numbers. CI, confidence interval; CR, complete response; CVD, cyclophosphamide–vincristine–dacarbazine; PD, progressive disease; PFS, progression-free survival; PR, partial response; PRRT, peptide receptor radionuclide therapy; SD, stable disease. + Censored.

Table S2. Spearman rank correlation coefficients between the Ki-67 index and the individual GAPP components.

GAPP component	Spearman ρ with Ki-67 index
Histological pattern	0.07
Capsular or vascular invasion	0.13
Comedo necrosis	0.25
Catecholamine type	−0.27
Cellularity	0.31
Mitotic count	0.53
GAPP total score	0.49

Correlations were calculated between the continuous Ki-67 index and each GAPP component scored as an ordinal variable, as defined in Section 2.3 of the main text; the coefficients are those reported in Section 3.6. Correlations with the non-proliferative components were weak, whereas those with mitotic count and with the GAPP total score were moderate. The Ki-67 index is itself one of the six components of the GAPP score, so its correlation with the GAPP total score is in part structural and should not be read as independent corroboration. GAPP, Grading system for Adrenal Pheochromocytoma and Paraganglioma.

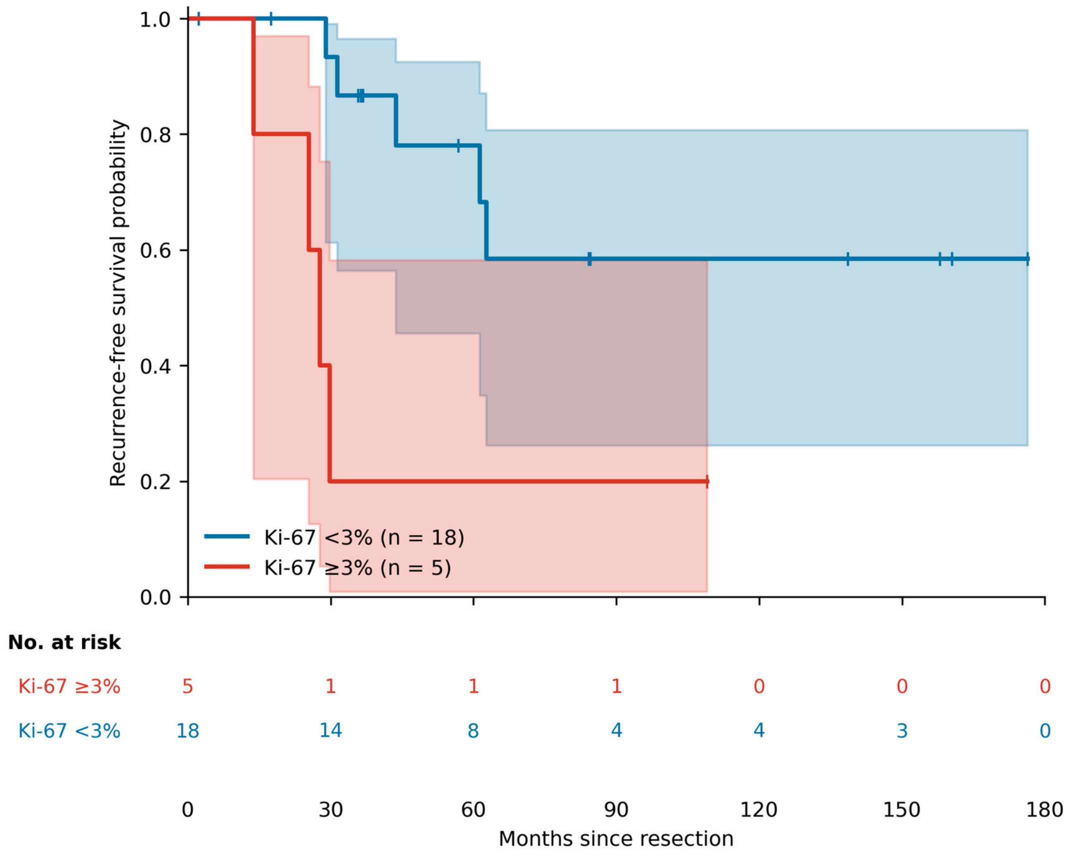

Figure S1. Kaplan–Meier estimates of recurrence-free survival according to the Ki-67 index, restricted to the 23 resected sympathetic (extra-adrenal) paragangliomas (9 events).

Tick marks denote censored observations and shaded bands are 95% confidence intervals. Only 5 patients had a Ki-67 index ≥3%, of whom 1 remained at risk beyond 30 months and none beyond 105 months; the curves in that stratum therefore rest on very few observations and are provided for transparency rather than for formal inference. The corresponding hazard ratio, 95% confidence interval and log-rank P value for this subgroup are reported in Section 3.6 of the main text.
